# Preoperative estimate of natural ureteral length based on computed tomography and/or plain radiography

**DOI:** 10.1038/s41598-021-91658-6

**Published:** 2021-06-09

**Authors:** Jen-Ting Hsu, Jen-Shu Tseng, Marcelo Chen, Fang-Ju Sun, Chien-Wen Chen, Wun-Rong Lin, Pai-Kai Chiang, Allen W. Chiu

**Affiliations:** 1 Department of Urology, MacKay Memorial Hospital, No. 92, Sec. 2, Zhongshan N. Rd., Zhongshan Dist., Taipei City, 10449 Taiwan, ROC; 2 Department of Radiology, MacKay Memorial Hospital, No. 92, Sec. 2, Zhongshan N. Rd., Taipei City, 10449 Taiwan, ROC; 3 Mackay Medical College, No.46, Sec. 3, Zhongzheng Rd., Sanzhi Dist., New Taipei City, 252 Taiwan, ROC; 4 Mackay Junior College of Medicine, Beitou District, Nursing, and Management, No.92, Shengjing Road, Taipei City, 11272 Taiwan, ROC; 5 School of Medicine, National Yang Ming Chiao Tung University, No.145, Zhengzhou Rd., Datong Dist., Taipei City, 10341 Taiwan, ROC; 6 Institute of Biomedical Informatics, National Yang Ming Chiao Tung University, No.155, Sec.2, Linong Street, Taipei, 112 Taiwan, ROC

**Keywords:** Ureter, Ureter

## Abstract

To predict natural ureter lengths based on clinical images. We reviewed our image database of patients who underwent multiphasic computed tomography urography from January 2019 to April 2020. Natural ureteral length (UL_CTU_) was measured using a three-dimensional curved multiplanar reformation technique. Patient parameters including age, height, and height of the lumbar spine, the index of ureteral length using kidney/ureter/bladder (KUB) radiographs (C-P and C-PS) and computed tomography (UL_CT_) were collected. UL_CTU_ correlated most strongly with UL_CT_. R square and adjusted R square values from multivariate regression were 0.686 and 0.678 (left side) and 0.516 and 0.503 (right side), respectively. UL_CTU_ could be estimated by the regression model in three different scenarios as follows:

- UL_CT_ + C-P
  - UL_CTUL_ = 0.405 $\times$ UL_CTL_ $+$ 0.626 $\times$ C-P_L_ – 0.508 cm
  - UL_CTUR_ = 0.558 $\times$ UL_CTR_ $+$ 0.218 $\times$ C-P_R_ + 6.533 cm
- UL_CT_
  - UL_CTUL_ = 0.876 $\times$ UL_CTL_ $+$ 6.337 cm
  - UL_CTUR_ = 0.710 $\times$ UL_CTR_ $+$ 9.625 cm
- C-P
  - UL_CTUL_ = 0.678 $$\times$$ C-P_L_ $$+$$ 4.836 cm
  - UL_CTUR_ = 0.495 $$\times$$ C-P_R_ $$+$$ 10.353 cm

We provide equations to predict UL_CTU_ based on CT, KUB or CT plus KUB for different clinical scenarios. The formula based on CT plus KUB provided the most accurate estimation, while the others had lower validation values but could still meet clinical needs.

UL_CT_ + C-P
  - UL_CTUL_ = 0.405 $\times$ UL_CTL_ $+$ 0.626 $\times$ C-P_L_ – 0.508 cm
  - UL_CTUR_ = 0.558 $\times$ UL_CTR_ $+$ 0.218 $\times$ C-P_R_ + 6.533 cm

UL_CTUL_ = 0.405 $\times$ UL_CTL_ $+$ 0.626 $\times$ C-P_L_ – 0.508 cm

UL_CTUR_ = 0.558 $\times$ UL_CTR_ $+$ 0.218 $\times$ C-P_R_ + 6.533 cm

UL_CT_
  - UL_CTUL_ = 0.876 $\times$ UL_CTL_ $+$ 6.337 cm
  - UL_CTUR_ = 0.710 $\times$ UL_CTR_ $+$ 9.625 cm

UL_CTUL_ = 0.876 $\times$ UL_CTL_ $+$ 6.337 cm

UL_CTUR_ = 0.710 $\times$ UL_CTR_ $+$ 9.625 cm

C-P
  - UL_CTUL_ = 0.678 $$\times$$ C-P_L_ $$+$$ 4.836 cm
  - UL_CTUR_ = 0.495 $$\times$$ C-P_R_ $$+$$ 10.353 cm

UL_CTUL_ = 0.678 $$\times$$ C-P_L_ $$+$$ 4.836 cm

UL_CTUR_ = 0.495 $$\times$$ C-P_R_ $$+$$ 10.353 cm

## Introduction

Ureteral stenting is now a fundamental and integral part of urologic surgery^1^, and it is commonly used to relieve ureteral obstructions and maintain patency^2–4^. Although its value and utility are clear, ureteral stenting is associated with significant stent-related symptoms^5,6^, and up to 80% of stented patients experience adverse events such as bladder pain and/or lower urinary tract symptoms^7–9^.

Discomfort and pain have been shown to increase with the diameter of the stent^10^. In addition, many studies and a recent review have shown that an excessively long stent, especially when the distal coil crosses the midline, may be associated with more urinary tract symptoms^11–13^. Several factors have been investigated with regards to their effects on stent-related symptoms and determining the appropriate ureteral length has been shown to be of significant importance^13–15^.

Although intraoperative direct ureteral length measurement has been shown to provide the most accurate ureteral length^16,17^, it has some disadvantages. Retrograde measurements require additional fluoroscopy, which is not available in some operation rooms or in the standard setting of regular ureteroscopic surgery. In addition, there is the risk of radiation exposure, not only to the patients, but also to the operating room staff. Retrograde catheterization for ureter length measurement has the added risk of iatrogenic trauma. Therefore, a more practical tool that is noninvasive, quick to perform and can be used to estimate ureteral length preoperatively is urgently needed.

We reviewed our image database for cases who underwent multiphasic computed tomography (CT) urography from January 2019 to April 2020. All of the images were reconstructed from stacked axial planes, and natural ureteral lengths were measured using a three-dimensional curved multiplanar reformation technique. We performed CTU measurements of non-diseased ureter as it resembles the ureter in its “natural” condition. In cadaveric studies, the measured ureters lack the physiologic smooth muscle tone. The retrograde method is usually used to measure the length of a diseased ureter (not in the “natural” condition), which is generally more flaccid or distended due to chronic obstruction.

The predictive factors for ureteral length included body height, height of the lumbar spine (L-spine), and kidney/ureter/bladder (KUB) radiographs and CT parameters. Statistical analysis was used to identify appropriate predictors, and equations to preoperatively estimate ureteral length in common clinical scenarios were derived, including KUB only, CT only, and KUB plus CT.

## Materials and methods

This retrospective study was conducted from January 2019 to April 2020. We enrolled patients who underwent CT urography at Mackay Memorial Hospital, a tertiary referral center in Northern Taiwan. Patients with diseased ureters were excluded on the basis of the following criteria: presence of renal abnormalities such as hydronephrosis, inflammatory lesions, renal cysts, renal tumors and congenital urological diseases; ureteral abnormalities such as urolithiasis, hydroureter, ureteral stent placement, duplication of ureter, upper tract urothelial carcinoma and ureteritis. Patients with filling defects in the ureter and bladder mass were also excluded.

### Measurement of ureteral length

Ureteral length was measured using multidetector computed tomography (MDCT). The MDCT scanners were a Siemens Somatom definition AS 64 and Siemens Somatom definition Flash 256, both of which were set to the following parameters: detector collimation: 64 × 0.6 mm; helical pitch: 1.0, section thickness/interval: 1 mm/1 mm; 120 kVp/250 mAs.

Images were reconstructed from stacked axial planes, showing the course of the ureter in a coronal plane. Using the curved multiplanar reformation technique, we could manipulate the image to tilt, rotate or view the ureter in various directions. Ureteral length (UL_CTU_) was measured by tracing from the ureteral pelvic junction (UPJ) to ureteral vesical junction (UVJ).

**Figure 1:**
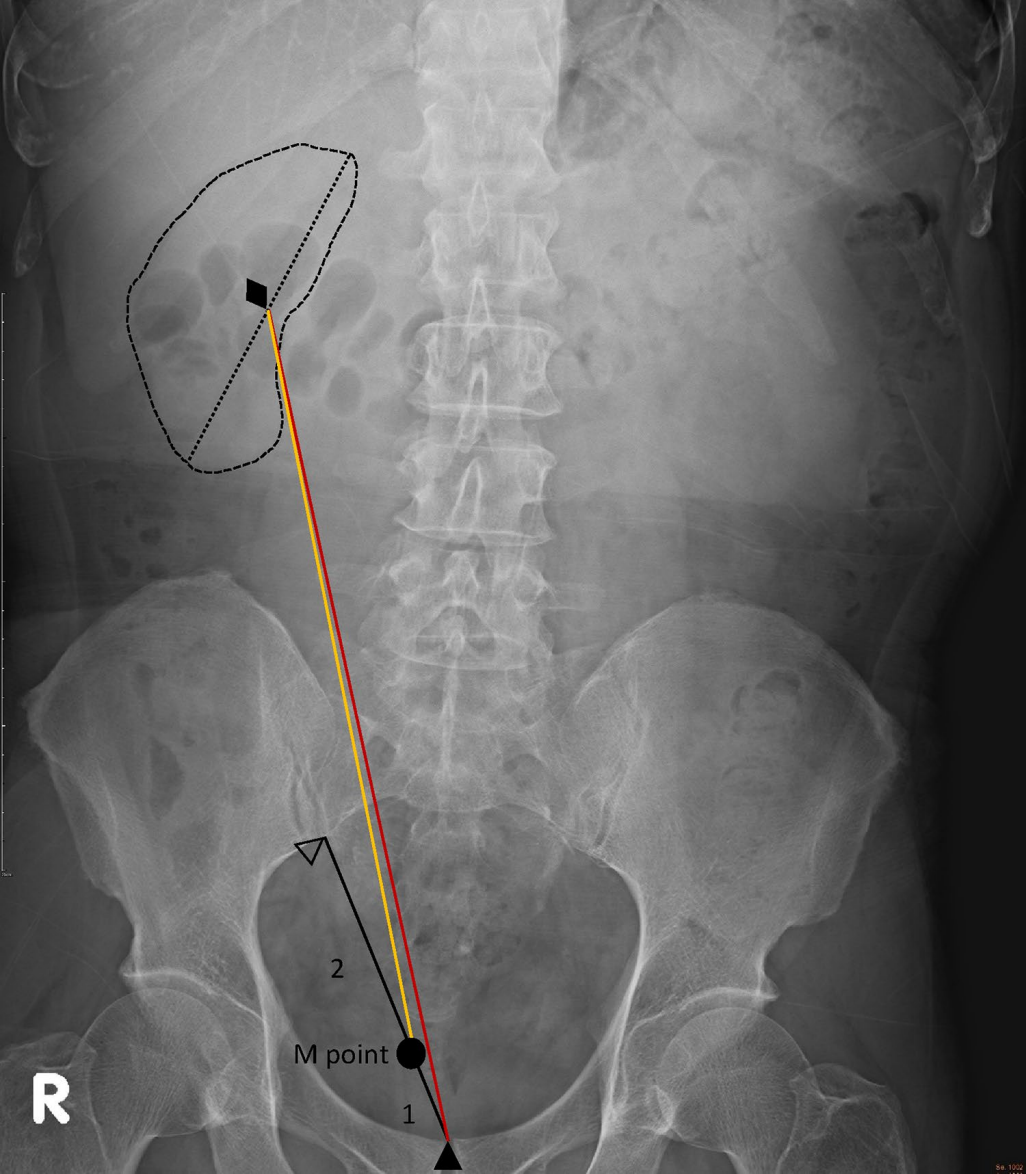
Measuring the length of C-P and C-PS. We took the point landing on the line from midpoint of superior margin of the pubic symphysis to the lower point of sacroiliac joint (P-S) as M point. M point was on one third the length of P-S closer to superior margin of pubic symphysis. C-PS was the length from central kidney point to M point. C-P was the length from central kidney point to superior margin of pubic symphysis. ♦: The central kidney point, defined as the midpoint of the long axis of kidney. ∆: The lower point of sacroiliac joint. ▲: The midpoint of superior margin of the pubic symphysis.

We chose two different methods to estimate ureteral length by using KUB radiography. KUB films were standardized at maximum inspiration and supine position. We took the point landing on the line from the midpoint of the superior margin of the pubic symphysis to the lower point of the sacroiliac joint (P-S) as the M point. The M point was one third the length of the P-S closer to the superior margin of the pubic symphysis. We defined the central kidney point as the midpoint of the long axis of the kidney. The index in our study was the length from the central kidney point to the M point (C-PS). The other index we measured on KUB radiography was the length from the central kidney point to the superior margin of the pubic symphysis (C-P). (Fig. 1).

We used axial reconstructions to measure the ureteral length on CT (UL_CT_)_._ UL_CT_ was determined by calculating the number of slices between the UPJ and UVJ. The UL_CT_ was calculated by multiplying the slice thickness and the number of slices between the UPJ and UVJ. The height of the lumbar spine was measured on CT.

### Statistical analysis

We separated the left and right ureter into two groups for statistical analysis. Clinical data were analyzed using descriptive statistics. Means with standard deviations (SDs) were used to summarize continuous variables, and percentages were used for categorical variables. The Student’s t-test was used to compare age, height, height of the lumbar spine, C-P, C-PS, UL_CT_ and UL_CTU_ between the left and right side. Pearson’s χ^2^ test or Fisher’s exact test was used for cells with a frequency of 5 or fewer, for categorical variables. We randomly assign 80% of the data for further Pearson correlation and regression analysis, and the rest of the data were used for validation. Relationships between variables were assessed using Pearson correlation analysis. A *P* value < 0.05 was defined as being statistically significant. We chose variables with correlation coefficients > 0.4 in both sides, considered to be a moderate correlation, for multiple regression analysis. Due to statistical collinearity between C-P and C-PS, defined as a variance inflation faction (VIF) > 10, we chose C-P instead of C-PS for multiple regression analysis if the correlation coefficient of C-P was higher. Three linear equations based on CT, KUB and CT plus KUB were derived from our regression models. Internal validation was tested for each regression model with 20% of our data. All statistical analyses were performed using R-4.0.2 statistical software.

### Ethics approval

The present study, including its research protocols and data collection, were approved by the Mackay Memorial Hospital Institutional Review Board. IRB No. 19MMHIS343e.

### Informed consent from patients

The study involved insensitive topics. MMH IRB No. 19MMHIS343e approved waiver of informed consent to the study.

## Result

A total of 165 ureters (87 left and 78 right) were included. Table 1 shows the patients’ demographic data of the two groups. The mean age was 60.6 years. The mean left UL_CTU_ (UL_CTUL_) was 25.4 $\pm$ 2.3 cm and the right UL_CTU_ (UL_CTUR_) was 24.8 $\pm$ 2.3 cm. The mean left C-P (C-P_L_) was 30.4 $\pm$ 2.3 cm and the right C-P (C-P_R_) was 29.2 $\pm$ 2.7 cm. The mean left C-PS (C-PS_L_) and right C-PS (C-PS_R_) were 26.8 $\pm$ 2.2 cm and 25.5 $\pm$ 2.6 cm, respectively, and the mean left UL_CT_ (UL_CTL_) and right UL_CT_ (UL_CTR_) were 21.8 $\pm$ 2.0 cm and 21.3 $\pm$ 2.3 cm, respectively.

**Table 1:** Patient characteristics and demographic data.

	Left	Right	value
N	87	78	
Age	60.6		0.85
**Gender**			
Female	31 (36.0)	30 (38.5)	0.87
Male	56 (64.0)	48 (61.5)	
Height	1.6 (0.1)	1.6 (0.1)	0.2
L-spine (cm)	15.5 (1.0)	15.4 (1.0)	0.37
C-P (cm)	30.4 (2.3)	29.2 (2.7)	0.004
C-PS (cm)	26.8 (2.2)	25.5 (2.6)	0.003
UL_CT_ (cm)	21.8 (2.0)	21.3 (2.3)	0.15
UL_CTU_ (cm)	25.4 (2.3)	24.8 (2.3)	0.088

Age is presented as number only. Other values are presented as mean with (standard deviation or percentage in gender). ULCTU, ureteral length measured with curved multiplanar reformation (MPR) technique, by tracing ureter from ureteral pelvic junction (UPJ) to ureteral vesical junction (UVJ).

UL_CTU_ was most strongly correlated with UL_CT_, with Pearson coefficients of 0.749 in the left group and 0.690 in the right group. C-P, C-PS and UL_CT_ had Pearson coefficients greater than C-PS_L_ and 0.4. Both coefficients of C-P_L_ (0.690) and C-P_R_ (0.570) were higher than C-PS_L_ (0.657) and C-PS_R_ (0.547). Age, height and L-spine were weakly correlated with UL_CTU_ (Table 2).

**Table 2:** The results of Pearson correlation analysis.

	UL_CTUL_		UL_CTUR_
Age	0.222	Age	0.126
Height	0.414	Height	0.383
L-spine	0.347	L-spine	0.215
C-P_L_	0.690	C-P_R_	0.570
C-PS_L_	0.657	C-PS_R_	0.547
UL_CTL_	0.749	UL_CTR_	0.690

The subscript L and R is represented with left side and right side, respectively. C-P and C-PS, length measured in KUB radiography. UL_CT_, length measured in axial CT.

In univariate regression analysis, the beta coefficients of UL_CTL_ and UL_CTR_ were 0.876 and 0.710, respectively. The beta coefficients of C-P_L_ and C-P_R_ were 0.678 and 0.495, respectively, and the beta coefficients of multivariate regression were 0.405 (UL_CTL_), 0.558 (UL_CTR_), 0.626 (C-P_L_) and 0.218 (C-P_R_), respectively. The constants were − 0.508 (left side) and + 6.522 (right side). The R square and adjusted R square values from multivariate regression were 0.686 and 0.678 (left side), and 0.516 and 0.503 (right side), respectively (Table 3).

**Table 3:** The results of regression and validation analyses.

	Univariate regression			Multivariate regression			
	B	T	*P*	B	T	*P*	VIF
UL_CTL_	0.876	10.433	< 0.001	0.405	2.597	0.013	
C-P_L_	0.678	8.799	< 0.001	0.626	5.871	< 0.001	33.528
C-PS_L_	0.689	8.044	< 0.001				32.748
Con				− 0.508	− 0.259	0.797	
				R square: 0.686, Adjusted R square: 0.678			

	Univariate regression			Multivariate regression			
	B	T	*P*	B	T	*P*	VIF
UL_CTR_	0.710	8.300	< 0.001	0.558	5.447	0.003	
C-P_R_	0.495	6.050	< 0.001	0.218	2.518	0.014	46.262
C-PS_R_	0.484	5.698	< 0.001				43.896
Con				6.533	3.025	0.718	
				R square: 0.516 Adjusted R square: 0.503			

The subscript L and R is represented with left side and right side, respectively. C-P and C-PS, length measured in KUB radiography. UL_CT_, length measured in axial CT. Con is the constant of regression.

UL_CTU_ could be estimated by equations derived from the regression model in three different scenarios as follows:

- UL_CT_ + C-P (measured with CT plus KUB)
  - UL_CTUL_ = 0.405 $\times$ UL_CTL_ $+$ 0.626 $\times$ C-P_L_ – 0.508 (cm; validation: 0.8861)
  - UL_CTUR_ = 0.558 $\times$ UL_CTR_ $+$ 0.218 $\times$ C-P_R_ + 6.533 (cm; validation: 0.7669)
- UL_CT_ (measured with CT only)
  - UL_CTUL_ = 0.876 $\times$ UL_CTL_ $+$ 6.337 (cm; validation: 0.8772)
  - UL_CTUR_ = 0.710 $\times$ UL_CTR_ $+$ 9.625 (cm; validation: 0.7626)
- C-P (measured with KUB only)
  - UL_CTUL_ = 0.678 $\times$ C-P_L_ $+$ 4.836 (cm; validation: 0.7344)
  - UL_CTUR_ = 0.495 $\times$ C-P_R_ $+$ 10.353 (cm; validation: 0.6168)

## Discussion

Ureteral stents were introduced in late 1940 and quickly became one of the most important devices in the field of urology^4^. However, ureteral stenting has both pros and cons. Adverse events such as bladder pain, frequency, urgency, and flank soreness have been reported in nearly 80% of patients receiving stents^14^. Consequently, means to decrease these stent related symptoms have been investigated^18^. The elasticity of stent material, coiling type, stent diameter and length have all been related to these symptoms, of which excess stent length is the mostly discussed factor. Choosing an appropriate stent has become an important part of clinical practice. In the past, height was used as a common and straightforward parameter to predict ureteral length. However, many reports have shown that height does not predict ureter length accurately^19,20^. Contemporarily, intraoperative direct measurement is the most accurate way to measure ureteral length^21^. However, additional procedure, prolonged operation duration, excess radiation exposure, and repetitive instrumentation limit its clinical utility. Preoperative prediction is a much more practical approach to meet clinical needs. Various prediction tools have been introduced based on preoperative images. However, these predictors have had their own limitations. Taguchi et al. showed the better prediction efficacy of KUB-based measurements (C-P) compared to CT-based measurements (P–V), However, Kawahara et al. compared body height, body surface, several measurements by intravenous urography, and CT-based measurements, and concluded that CT outperformed the other factors. The same study group developed a nomogram using five preoperative characteristics including age, side, sex, IVU measurements, and CT calculation. Jung et al. reported equations to predict ureteral length by using the length between the UVJ and UPJ on CT, and standing/sitting body height^22^. In our study, the CT-based measurement had a better predictive value, which is compatible with previous studies.

The biggest disadvantage of previous reported prediction tools has been the need for CT measurements. Despite its known value in the diagnosis of urolithiasis, CT images are not always available^23^. Diagnosis with plain radiography is more common as it is faster, requires less radiation exposure, and has a lower cost, even though it cannot detect radiolucent stones^24^. In real-world clinical situations, there would be KUB only in many cases and only a few cases would have both CT and KUB or IVU. The first part of our formula was developed for patients who received both CT and KUB studies, which had the highest value of validation. In cases of CT or KUB only, we provided other alternative equations which had lower validations but could still meet clinical needs. Validation in our study also revealed a high correlation between predictive value and actual UL_CTU_, especially in the CT + KUB regression model, with 0.8861 (left side) and 0.7669 (right side).

Previous studies have used formulas derived from height to estimate ureteral length. Nevertheless, most databases in these studies were based on Caucasians, and the average height of Caucasians is taller than Asians^25^. Therefore, racial and regional differences in ureteral length should be taken into account. Our study design provides a quick and favorable clinical method with high validation to build equations to estimate natural ureteral length. Further studies in applying our method or equations in patients of different regions are needed.

There are still some limitations to our study. First, we focused on predicting the natural ureter length instead of choosing the stents directly. The correlation between calculated ureter length and proper stent size needs further investigations. Second, this is a retrospective study. In the future, we would like to apply our study clinically and plan a prospective study including preoperative prediction, actual stent insertion and post-operative ureteral stent-related symptom questionnaires. We believe this would be a more practical way to assess proper ureteral stenting and prevent patients from stent-related symptom. There would be more extensive applications such as the use in the planning of bioengineered ureter replacement in the future where the amount of tissue could be estimated by the length to be replaced.

## Conclusion

We provide equations to predict UL_CTU_ based on CT, KUB or CT plus KUB for different clinical scenarios. The formula based on CT plus KUB provided the most accurate estimation, while the others had lower validation value but could still meet clinical needs.

## Data Availability

Records and data pertaining to this case are in the patient’s secure medical records in Mackay Memorial Hospital. The datasets generated during and/or analysed during the current study are available from the corresponding author on reasonable request.
